# Authenticity Preservation with Histogram-Based Reversible Data Hiding and Quadtree Concepts

**DOI:** 10.3390/s111009717

**Published:** 2011-10-13

**Authors:** Hsiang-Cheh Huang, Wai-Chi Fang

**Affiliations:** 1 Department of Electrical Engineering, National University of Kaohsiung, 700 University Road, Kaohsiung 811, Taiwan; 2 Department of Electronics Engineering, National Chiao-Tung University, 1001 Ta-Hsueh Road, Hsinchu 300, Taiwan; E-Mail: Dr.WFang@gmail.com

**Keywords:** reversible data hiding, histogram, quadtree, imperceptibility, capacity, side information

## Abstract

With the widespread use of identification systems, establishing authenticity with sensors has become an important research issue. Among the schemes for making authenticity verification based on information security possible, reversible data hiding has attracted much attention during the past few years. With its characteristics of reversibility, the scheme is required to fulfill the goals from two aspects. On the one hand, at the encoder, the secret information needs to be embedded into the original image by some algorithms, such that the output image will resemble the input one as much as possible. On the other hand, at the decoder, both the secret information and the original image must be correctly extracted and recovered, and they should be identical to their embedding counterparts. Under the requirement of reversibility, for evaluating the performance of the data hiding algorithm, the output image quality, named imperceptibility, and the number of bits for embedding, called capacity, are the two key factors to access the effectiveness of the algorithm. Besides, the size of side information for making decoding possible should also be evaluated. Here we consider using the characteristics of original images for developing our method with better performance. In this paper, we propose an algorithm that has the ability to provide more capacity than conventional algorithms, with similar output image quality after embedding, and comparable side information produced. Simulation results demonstrate the applicability and better performance of our algorithm.

## 1. Introduction

**Figure 1.**
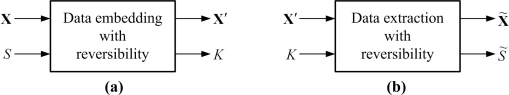
**(a)** The structure for reversible data embedding. **(b)** The structure for reversible data extraction.

Authentication preservation with sensors and related applications have emerged as an important research field in recent years [1–4]. Among the authentication schemes, reversible data hiding can be considered as an effective means in this field. Reversible data hiding, also referred to as lossless data hiding or reversible watermarking, has been a newly developed branch and an interesting watermarking research topic [5,6]. Figure 1 outlines the scenario presented in this paper. Suppose that the cover media for secret transmission of data are the digital images, denoted by **X** in Figure 1(a). With the term “reversibility”, at the encoder, the user-defined secret, denoted by *S* in Figure 1(a), should be embedded into the cover image **X** by the reversible data hiding method proposed by algorithm designers. After accomplishing the embedding process, the image containing the secret information, denoted by **X′**, and the side information for making decoding possible, denoted by *K*, should be transmitted to the decoder. The size of *S* can be as large as possible, while the size of *K* should be as small as possible. And at the decoder in Figure 1(b), by using the received **X′** and *K*, the reconstructed image **X̃** and the extracted secret *S̃* can be recovered. Due to reversibility, **X̃** and *S̃* should be identical to their counterparts of **X** and *S*, respectively. Therefore, how to design an effective algorithm for reversible data hiding is an interesting task in both research and application.

There are requirements for designing a good reversible data hiding algorithm [7–9]. Four major requirements are: (a) the output image quality, called the imperceptibility, (b) the number of bits that can be hidden into the cover image, named the capacity, (c) the side information that is necessary for performing data extraction at the decoder, and (d) the ease of implementation of the algorithm. And we observe that these requirements have some correlations or conflict with the others. For instance, embedding more capacity would lead to the more deterioration to the cover image, and hence the degradation result in imperceptibility of the output image. From the viewpoint of practical applications, less overhead is greatly required. Therefore, how to develop a reversible data hiding algorithm that can hide more capacity, produce the output image quality with acceptable imperceptibility, and generate as little side information as possible are much required, and these are the major contributions of this paper.

This paper is organized as follows. In Section 2, due to the ease of implementation, we present fundamental descriptions of conventional histogram-based reversible data hiding algorithm. Then, in Section 3, for making good use of the characteristics of the cover image, we take the quadtree decomposition into account and look for the integration with the algorithm presented in Section 2. Simulation results are demonstrated in Section 4, which suggest the applicability of the algorithm and the integration proposed over conventional schemes. Finally, we conclude this paper in Section 5.

## 2. Descriptions of Conventional Histogram-Based Reversible Data Hiding

The concept of data hiding is to embed secret information relating to the copyright owner into original multimedia content, depicted in Figure 1. For reversible data hiding, the secret to be hidden, *S*, is first embedded into the cover image **X**. Later on, at the decoder, by performing the reverse of the information embedding process, both the embedded secret and the recovered image, which is exactly the same as the cover image, should be extracted. The main challenge of reversible data hiding is to develop an algorithm that is reversible, that is, all the inputs to the encoder in Figure 1(a), including the cover image **X** and hidden secret *S*, must be perfectly extracted at the decoder after delivery of the marked image **X′**. After completing the extraction procedure, the outputs of the extractor in Figure 1(b) must be identical to the inputs of encoder in Figure 1(a), or **X̃** = **X** and *S̃* = *S*.

For the implementation of reversible data hiding algorithms, reversible data hiding can be categorized into three major branches. The first one is by modifying the histogram of original image for embedding the user-defined information [8], the second one is called the difference expansion (DE) scheme [9], and the third one is by the replacement of the least significant bits (LSB) of the original image. Characteristics of the three schemes are briefly discussed as follows.

For the histogram-based scheme to be addressed shortly in Section 2.1, for making data embedding possible, only a portion of the histogram needs to be moved. It implies that no calculation is necessary in the data embedding process. Due to the ease of implementation, we will focus on the histogram-based scheme in this paper.

For the DE scheme, the average and difference values between two consecutive pixels need to be calculated. Also, for data embedding, multiplication of the difference value is required, and that’s the meaning of “difference expansion” originated. Besides, the quality of the output image can hardly be guaranteed with the DE scheme even though a large embedding capacity can be expected. Most important of all, some pixel pairs may be unsuitable for making data hiding possible because the difference between two consecutive pixels may be large, which would cause an overflow problem after embedding with difference expansion. These unsuitable positions for data hiding, called the location map, need to be recorded for correct extraction of data at the decoder. Location map should be delivered to the decoder for data extraction and original recovery, which is regarded as the side information for reversible data hiding. Based on the characteristics of original images, the size of location map may grow to some considerable amount, which would limit the performances with the DE scheme.

Finally, for the LSB-replacement scheme, it is one of the simplest schemes for reversible data hiding. By hiding the secret data into the LSB of the original image, reversible data hiding can be completed. Because papers focusing on this topic were a bit outdated, mostly published around ten years ago, we omit the comparisons with this scheme.

Due to the ease of implementation, we concentrate on the histogram-based scheme in this paper to realize the application of our method.

### 2.1. Histogram-Modification for Reversible Data Embedding and Extraction

Histogram-modification scheme for data embedding is adopted from [8], which can be described with the following Steps.

**Figure 2.**
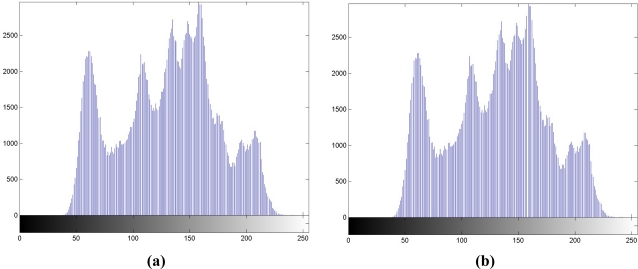

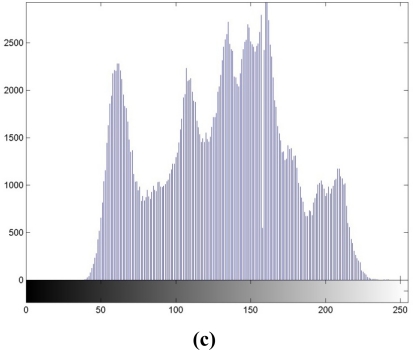
**(a)** Histogram of Lena. **(b)** After performing Step 3. Correlation coefficient = 0.9957. **(c)** After performing Step 4. Correlation coefficient = 0.9958.

Step 1. *Histogram generation.* We generate the histogram of **X** in Figure 2(a) first. The luminance with the maximal occurrences in histogram is labeled as “max point,” while that with no occurrence is labeled as “zero point.” Without loss of generality, we assume that the luminance of the zero point is larger than that of the max point. The luminance values of “max” and “zero” points, each is represented by 1 byte (or 8 bits), are treated as overhead or side information, *K*. Hence, a total of 16 bits should be transmitted to the receiver for data extraction.
Step 2. *Range selection.* The range of luminance values between max and zero points is recorded in the histogram.
Step 3. *Luminance addition in selected range.* In the region between max and zero points recorded in Step 2, luminance values in the selected range are all increased by 1, and we can regard the selected region is shifted to the right by 1. The resulting histogram is depicted in Figure 2(b), and it has high correlations with Figure 2(a), which has the correlation coefficient of 0.9957.
Step 4. *Data embedding.* For the embedding of user-defined bitstream *S* in Figure 1(a), if the watermark bit is ‘1,’ the luminance value keeps unchanged; if the watermark bit is ‘0’, it is decreased by 1. Figure 2(c) depicts the histogram with this step, and it is also highly correlated with Figure 2(a), with the correlation coefficient of 0.9958.

In extracting both the hidden data and cover image, the following steps should be executed accordingly:

Step 1. *Locating of range.* With side information *K* in Figure 1(b), luminance values between the max and zero points are compared.
Step 2. *Data extract.* Every pixel in **X′** in Figure 1(b) is scanned and examined sequentially to extract the data bits. Performing the reverse procedure to Step 3 of the embedding procedure, extracted data *S̃* is obtained.
Step 3. *Original image recovery.* By moving the histogram into its original form, the content *X̃* is recovered. Only the max point is required.
Step 4. *Reversibility validation.* After data extraction, we need to validate whether the outputs of the decoder and the inputs of the encoder are exactly the same. That is, **X̃** = **X**, and *S̃* = *S*.

Performing data hiding is simply by shifting certain parts of the histogram of the image, and no calculation is required. Besides, the luminance values of the max and zero points play an important role for making reversible data hiding possible.

### 2.2. Advantages and Drawbacks of the Conventional Scheme

Among the requirements addressed in Section 1, the histogram-based reversible data hiding has the advantages of ease of implementation, guaranteed output image quality, and little side information produced [8]. On the contrary, the limited amount of embedding capacity is the major drawback for this algorithm. With the descriptions in Section 2.1, we observe the advantages of the histogram-based reversible data hiding, which are depicted as follows.

➢ Ease of implementation: The portion between max and zero points in the histogram is intentionally shifted to the right by one. No calculation for performing this procedure is necessary.
➢ Guaranteed imperceptibility: The mean squared error (MSE) between the original and output images would be at most 1.00 [8], meaning that the Peak Signal-to-Noise Ratio (PSNR) would be at least 48.13 dB.
➢ Little overhead: Only the luminance values of the max and zero points are required at the decoder for extracting the data and the original, meaning that 2 bytes (or 16 bits) of overhead is necessary, regardless of the size of original images.

On the contrary, the major drawback of the histogram-based scheme is described as follows.

➢ Limited amount of capacity: The embedding capacity is limited by the number of occurrences at the max point, and this value is directly affected by the characteristics of the original image. Suppose that the cover image in grayscale has the size of *M* × *N* pixels, and each pixel is represented by 1 byte. Under the worst case when the occurrences of max points are the same for every luminance value, meaning that the histogram is flat, it represents the uniform distribution between 0 and 255, and the capacity for embedding is only *M*×*N*÷256 bits. This implies that embedding capacity is only 0.049% of the filesize of cover image.

From the observations above, we are able to take the characteristics of the original image into consideration, and try to increase the capacity at the expense of somewhat degraded quality of the output image. Side information should be comparable to that of the conventional scheme. Therefore, we employ the concept of quadtree decomposition for the implementation of histogram-based reversible data hiding algorithm.

### 2.3. Comparisons with Related Schemes

Several histogram-based reversible data hiding schemes to improve the performance of the conventional scheme in [8] have been proposed recently. We choose two closely relating schemes with the histogram-based scheme in [10] and [11] to make comparisons.

In [10], authors modified the scheme in [8] to increase the embedding capacity. The authors proposed to use the location map to record the maximum and minimum points in the histogram of original image, and reserve some spaces in the location map to mark the coordinates of the max and zero points of the histogram. The method in [10] has the advantages of increased embedding capacity and guaranteed output image quality of at least 48.13 dB. However, the induction of location map would reduce the capacity to some extent. In short, results in [10] outperform those in [8].

In [11], its authors took the human visual system (HVS) into consideration. Authors employed a causal window, 3 × 3 or 5 × 5 respectively, to calculate the predicted pixel value for data embedding based on HVS. By modifying the difference between the predicted pixel value and the actual pixel value, secret bits can be embedded. Resulting difference values should lie within the range of just noticeable distortion (JND). The method in [11] has the advantages of a much more amount of embedding capacity for hiding the secret bits into unnoticeable regions. However, the calculation of HVS adds more burden to implementation, and severe degradation in output image quality can be observed objectively.

With the comparisons above, in this paper, we propose the histogram-based reversible data hiding with quadtree decomposition in order to look for the better performances and practical applications. Unlike the use HVS in [11], we employ quadtree decomposition to hide the secret bits into appropriate regions after decomposition, and output image quality can be guaranteed objectively. After data hiding, secret bits would be imperceptible subjectively. Most important of all, reversibility of the data hiding algorithm must be retained for the application of authenticity preservation.

## 3. Histogram-Based Reversible Data Hiding with Quadtree Decomposition

**Figure 3.**
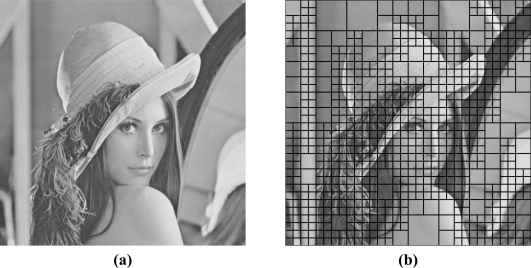
**(a)** The Lena test image. **(b)** After quadtree decomposition of Lena.

The quadtree decomposition analyzes the characteristics of cover images. It classifies the contents of cover images into smooth or active regions by using different block sizes. With the ease of representation in Figure 3, it decomposes the cover image into larger to smaller square blocks based on the smoothness of that region; the larger blocks denote the smooth regions, while the smaller ones denote the active regions. Figure 3(a) represents the original image Lena with the size of 512 × 512, while Figure 3(b) denotes the corresponding quadreee decomposition with Figure 3(a). We consider each square block with the different size as a small image, and employ the histogram-based scheme in Section 2 to look for the better performance both in embedding capacity and in output image quality.

With the configuration that every block is regarded as a small image, if the cover image is composed of *N* blocks with different sizes after quadtree decomposition, the side information for the whole image will grow to 2*N* bytes because each block corresponds to 2 bytes of overhead. In addition, the size of each block should also be recorded for making reversible decoding possible. And the sizes can be regarded as another source of side information. Therefore, how to effectively represent the side information corresponding to quadtree decomposition should be carefully treated. With our method, the increase of the size of side information can be reduced, while keeping reversible data hiding possible.

### 3.1. The Three-Round Embedding Process

**Figure 4.**
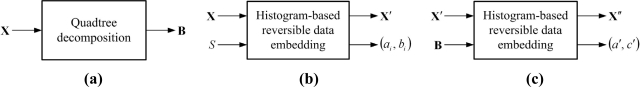
**(a)** Diagram for Round 1. **(b)** Diagram for Round 2. **(c)** Diagram for Round 3.

In conjunction with the conventional histogram-based reversible data hiding described in Section 2, we propose the three-round embedding process in Figure 4 with the characteristics of original image by using quadtree decomposition. The proposed scheme can be briefly outlined as follows.

**Round 1.** Perform the quadtree decomposition to the original image, **X**, and obtain different block sizes for the composition of original image. Find the luminance value of the zero point of the whole image. Save each block size and the luminance value of max point at the corresponding position as the block map (BM), **B**, depicted in Figure 4(a).
**Round 2.** Apply the histogram-based reversible data hiding for embedding information, *S*, to each block in the original image **X**. Output the image containing *S*, and call it **X′**. We can directly employ Figure 1(a) in this Round, and represented by Figure 4(b).
**Round 3.** After completing data embedding in Round 2, we apply the histogram-based reversible data embedding again, and embed the block map **B** into **X′**. Call the output image of this round as **X″**. Transmit the side information *K* to the decoder, shown in Figure 4(c).

Here we go into more details in each of the three rounds. At Round 1, we demonstrate the procedure in Figure 4, with the original “Lena” image in Figure 3(a). We can easily find that after quadtree decomposition, based on the characteristics of original image, it can be decomposed into square blocks with sizes among 16 × 16, 32 × 32, and 64 × 64, in Figure 3(b). Homogeneity of each block of original image can be determined by a threshold *T*, which may be calculated by:

(1) $$\{\begin{array}{lll} (\text{max}\left(\text{block value}\right)-\text{min}\left(\text{block value}\right))\geq T & \Rightarrow & \text{Keep decomposition};\\ (\text{max}\left(\text{block value}\right)-\text{min}\left(\text{block value}\right))<T & \Rightarrow & \text{Stop.} \end{array}$$

We set *T* = *α*·255, *α* ∈ [0,1]. The value of *α* can be adjusted by the users. Thus, for smaller threshold values, more blocks will be produced, and the size of the block map **B** will grow accordingly.

Next, at Round 2 of Figure 4(b), each square block in Figure 3(b) can be regarded as a small image. From the descriptions in Section 2, after the embedding with histogram-based reversible data hiding, the two luminance values for max and zero points, namely, *a_i_* for the max point and *b_i_* for the zero point for the *i*-th block, respectively, and each can be represented by 8 bits, are served as the side information. Without loss of generality, we assume that *a_i_* < *b_i_*, ∀*i*. Under the extreme case when the original image is highly active, which implies more blocks, 16 × 16 each, would be necessary for decomposing the original image. Then, it would lead to $\frac{512}{16}\times \frac{512}{16}=1024$ blocks in total, and 1,024 × (8 + 8) = 16,348 bits of side information are produced.

**Figure 5.**
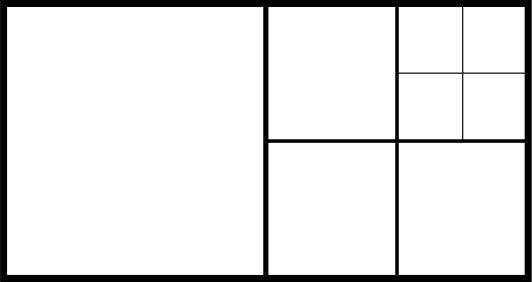
Depiction of 8 × 16 image with three-level quadtree decomposition.

Because only the value *a_i_* in Figure 3(b) is required for data extraction in each block, we set all the values of *b_i_* to be the luminance value at zero point of the whole image, *b*, to reduce the overhead. By doing so, at most 1,024 × 8 + 8 = 8,200 bits of side information of the block map **B** is produced. For the better representation the quadtree decomposition, approaches are the same in the method in [12,13] (called the quadtree partition, QP) as that in our methods (*i.e*., the block map **B**). Both employ the pyramidal hierarchy for data transmission. We can perform a brief depiction with the help of Figure 5. Suppose that an image with the size of 8 × 16 is partitioned by quadtree decomposition in Figure 5. The three-level decomposition of the block map **B** can be represented as follows:

➢ For the first level with the block size of 8 × 8, the block map **B** to be coded is ‘01’;
➢ For the second level with the block size of 4 × 4, the block map **B** to be coded is ‘0100’;
➢ For the third level with the block size of 2 × 2, the block map **B** to be coded is ‘0000’.

With the formation of **B**, quadtree decomposition can be included into the histogram-based reversible data hiding.

Finally, at Round 3, after embedding the user-defined information *S* at Round 2, histogram-based embedding should be performed on **X′**, and the max point in **X′**, *a′*, should be at least 8,200 in Figure 4(c). If the max point in **X′** is incapable of embedding 8,200 bits, we will search for luminance values *c′* < *a′*, such that the occurrences for both *a′* and *c′* are greater than 8,200. After the embedding of the block map **B** is performed, the two-byte side information, containing *a′* and *c′*, is transmitted to the decoder. That is, *K* = (*a′*, *c′*). Therefore, with our algorithm, very little overhead is needed for decoding. This amount of side information is comparable to those shown in literature.

### 3.2. The Three-Round Extraction Process

The goal for the extraction process is to recover both the original image **X** and the user-defined information *S* at the decoder, with the two-byte side information *a′* and *c′*. These are the reverse operations to the embedding process, and they can be outlined as follows.

**Round 1.** Perform histogram-based reversible data extraction on **X″** with the side information *K*. Reconstruct the block map **B** and obtain the luminance value of the max point for each block, *a_i_*.
**Round 2.** Reconstruct the image **X′**, which denote the original image **X** with the user-defined information *S*.
**Round 3.** Generate the user-defined information *S* and the original image **X** with the aid of **B** and the conventional histogram-based reversible data extraction.

By following the descriptions above, we can effectively hide the user-defined information *S* into the cover image **X** with high capacity and little overhead. At the decoder, after the reconstruction of the block map associated with quadtree decomposition, both the user-defined information and the cover image can be obtained. For verifying the reversibility of our algorithm, both the inputs to the encoder and the outputs to the decoder are compared by calculating the mean square error (MSE) of images and the bit-error rate (BER) between embedded and extracted information. When both the MSE and the BER values are 0.0, the image and the user-defined information at the encoder in Figure 1(a) and at the decoder in Figure 1(b) are identical, and it implies that the reversibility of the algorithm is verified.

## 4. Simulation Results

We perform the following simulations for the evaluation of our algorithm. Requirements are evaluated below in comparison with conventional algorithm, including:

➢ the output image quality, represented by PSNR, after hiding the user-defined data;
➢ the capacity, represented by bits, of the user-defined binary random data;
➢ the size of the overhead.

The first two requirements are easy for making comparisons. For the third one, we find that in Figure 3(b), the block sizes after performing quadtree decomposition are different. Therefore, the different block sizes compose of the block map **B** for making data embedding and extraction possible. In order to make fair comparisons, we divide the original image into blocks with the same sizes of 512 × 512, 256 × 256, 128 × 128, 64 × 64, 32 × 32, and 16 × 16, respectively.

**Figure 6.**
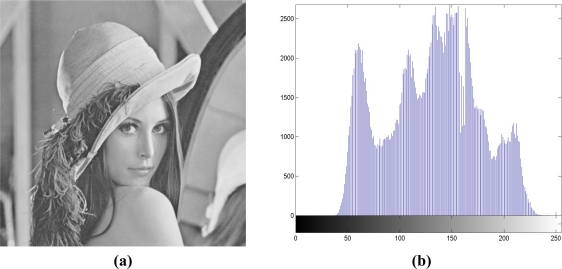
**(a)** The output image **X″** in Figure 3(c), with PSNR = 51.13 dB. **(b)** Corresponding histogram with **X″**. Correlation coefficient = 0.9808.

In contrast with quatree decomposition, these configurations have regular patterns, and only one block size needs to be included into the block map. Hence, except for the block size, only luminance of the max point in each block should be included in the block maps. On the contrary, with quadtree decomposition, we may expect the more overhead in the block map **B** because the side information of each block is composed of the block size and the luminance of max point. To make fair comparisons, even though the block map with quadtree decomposition is larger than the other cases, Round 3 of the embedding process comprises the block map even though some degradation in output image quality can be expected. To embed block map **B** in Section 3.1, in the Lena image in Figure 6, we choose *a′* = 159 and *c′* = 237 to serve as the side information, and the block map **B** can be embedded.

Table 1 depicts the comparisons between the image quality and embedding capacity under a variety of block sizes. For the block size of 512 × 512, we find that the capacity is 0.0113 bit/pixel (bpp), or 2,958 bits in the 512 × 512 image, with the PSNR of 53.54 dB. These serve as a reference corresponding to existing scheme in [8]. When the block size gets smaller, more capacity can be embedded at the expense of somewhat degraded quality. Because the image qualities under all the block sizes are larger than 48.13 dB, we can claim that these qualities are acceptable. With quadtree decomposition, the performances accessed by quality and capacity lie between the block sizes of 32 × 32 and 256 × 256. If we set the PSNR the same, we can see that with quadtree decomposition, our algorithm can hide more capacity than the 64 × 64 case. In Figure 6, we present the output after reversible data hiding and corresponding histogram for subjective evaluation. Figure 6(a) denotes the output **X″** of Figure 3(c), with the PSNR of 51.13 dB. Figure 6(b) depicts the output histogram, and it is also highly correlated with Figure 2(a), with the correlation coefficient of 0.9808. After embedding 14,303 random bits with our scheme or the capacity of 0.0546 bpp, the quality is still guaranteed.

Table 2 represents the simulation results with different test images. The image sizes are all 512 × 512 for making fair comparisons. Regarding to the image qualities, they are all more than 48.13 dB. Corresponding capacities are also provided, and we observe that capacities are highly correlated to the characteristics of original images, ranging from 0.0493 to 0.3651 bpp. Next, the increases for the quadtree decomposition over existing method are provided. For instance, for the Lena image, the capacities for the quadtree and the conventional methods [8] are 0.0546 and 0.0113 bpp, respectively. Therefore, we can easily calculate the increase in percentage by $\left(\frac{0.0546}{0.0113}-1\right)\times 100\%=284\%$. At the decoder, after decoding with the 2-byte side information (*a′*, *c′*), the block map **B** can be produced, and then both the cover image and user-defined information can be extracted. For verifying the reversibility of our algorithm, regarding to the image itself, we can see that all the mean square errors (MSE’s) are 0.00, meaning that the recovered images are identical to their counterparts. Besides, for user-defined information, we observe the bit error rates (BER’s) between the embedded and extracted ones are all 0.00%, meaning that they are identical. Therefore, from the data shown in the bottom two rows in Table 2, we prove that our data hiding algorithm can reach the goal of reversibility.

Table 3 makes performance comparisons with the results in [10] and those with our scheme. Method in [10] also employs the histogram-based scheme for performing reversible data hiding. Suppose that the input images are identical, both are the test image Lena with the size of 512 × 512. For the maximum number of capacity, they are 0.0204 and 0.0547 bit/pixel with the method in [10] and with our scheme, respectively, leading to the enhancement of $\left(\frac{0.0546}{0.0204}-1\right)\times 100\%=168\;\%$. With this capacity, the output image with our scheme obtains the better quality of 2.93 dB in difference. Because the MSE and BER values between the two schemes are 0.0, it implies that reversibility can be retained with the two schemes. In short, our scheme outperforms that in [10] with the statistics in Table 3.

**Figure 7.**
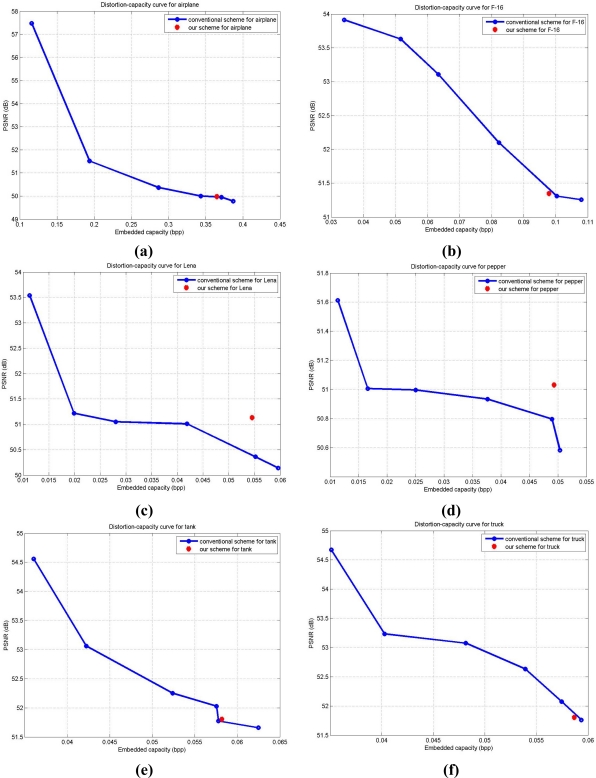
Distortion-capacity curves for the test images. Blue curves present the distortion-capacity curve with the modified scheme in [8], and the red dot denotes the result with our scheme. **(a)** airplane. **(b)** F-16. **(c)** Lena. **(d)** pepper. **(e)** tank. **(f)** truck.

Corresponding to the results represented in Table 2, we demonstrate the distortion-capacity performances in Figure 7 for the better understanding of the results. In Figure 7, the blue curve presents the distortion-capacity curve with the modified conventional scheme in [8], and the red dot denotes the result for quadtree decomposition with our scheme. We modify conventional scheme in [8] by using regular block sizes of 512 × 512, 256 × 256, 128 × 128, 64 × 64, 32 × 32, or 16 × 16, and treat each block as a small image for data embedding for making fair comparisons with the proposed scheme. In Figure 7(a,c–e) for airplane, Lena, pepper, and tank, respectively, proposed results outperform those with conventional scheme. For the F-16 and truck images in Figure 7(b,f), the modified conventional scheme performs a bit better than our scheme. This might be because the side information cancelled out the gain in the real capacity and corresponding output image quality. In short, the block map in quadtree decomposition is directly affected by the characteristics of cover image. The enhancement in capacity with our scheme might be balanced by the increase of size in block map. And we need to mention again that the output image quality among all the six images are all more than 48.13 dB, as we discussed earlier in Section 2.

## 5. Conclusions

In this paper, we have proposed a three-round reversible data hiding algorithm with the characteristics of the original image for authentication preservation with sensors. Quadtree decomposition is taken into account in order to increase the embedding capacity with acceptable quality of the output image. With our simulation results, we have obtained more embedding capacity, acceptable output image quality, and a comparable amount of side information produced. Most important of all, we have verified that the proposed algorithm can reach the goal of reversibility, which can make further authentications possible.

We have conducted simulations based on the easily implemented algorithm by modifying the histogram of original image. The other branch for reversible data hiding, based on modifying the differences between consecutive pixels [9], or the integration with DE, can also be taken into account for further improvement of our algorithm in the future.

## Figures and Tables

**Table 1.** Comparisons of test image Lena with conventional scheme.

**Block Size**	512 × 512	256 × 256	128 × 128	64 × 64	32 × 32	16 × 16	**Quadtree**
Image quality (dB)	53.54	51.22	51.03	51.02	50.36	50.14	51.13
Capacity (bit/pixel)	0.0113	0.0199	0.0280	0.0419	0.0552	0.0596	0.0546

**Table 2.** Comparisons of performances of test images, in alphabetical order, with conventional scheme.

**Test image**	**Airplane**	**F-16**	**Lena**	**Pepper**	**Tank**	**Truck**
Image size	512 × 512	512 × 512	512 × 512	512 × 512	512 × 512	512 × 512
Output image quality (dB)	49.97	51.34	51.13	51.03	51.80	51.81
Capacity (bit/pixel)	0.3651	0.0980	0.0547	0.0493	0.0582	0.0587
Increase in capacity over existing one (%)	214%	188%	284%	335%	61%	67%
MSE between original and recovered images	0.00	0.00	0.00	0.00	0.00	0.00
BER between embedded and extracted information	0.00%	0.00%	0.00%	0.00%	0.00%	0.00%

**Table 3.** Comparisons with [10] for test image Lena.

**Schemes**	**Results in [10]**	**Results with our scheme**
Image size id Lena	512 × 512	512 × 512
Output image quality (dB)	48.20	51.13
Capacity (bit/pixel)	0.0204	0.0546
Increase in capacity over [10] (%)	–	168%
MSE between original and recovered images	0.00	0.00
BER between embedded and extracted info	0.00%	0.00%
